# Microcirculatory impairment and increased arterial stiffness in pediatric Long COVID patients

**DOI:** 10.1007/s00431-026-06825-6

**Published:** 2026-03-16

**Authors:** Julie Boever, André Jakob, Clara Paetzold, Delphina Gomes, Lena T. Birzele, Katrin Baalmann, Nikolaus A. Haas, Claudia Nussbaum

**Affiliations:** 1 Department of Pediatric Cardiology and Pediatric Intensive Care, University Hospital, LMU Munich, Munich, Germany; 2 Division of Pediatric Infectious Disease, Department of Pediatrics, Dr. Von Hauner Children’s Hospital, University Hospital, LMU Munich, Munich, Germany; 3 Division of Neonatology, Department of Pediatrics, Dr. Von Hauner Children’s Hospital, University Hospital, LMU Munich, Munich, Germany

**Keywords:** Pediatric Long COVID, Microcirculation, Endothelial dysfunction, Arterial stiffness, Sidestream dark field imaging, Peripheral arterial tonometry

## Abstract

**Purpose:**

The exact pathogenesis of Long COVID remains unclear. Microvascular and endothelial dysfunction, established contributors to SARS-CoV-2-related conditions, appear to play a role in pediatric Long COVID.

**Methods:**

At the Children’s University Hospital of LMU Munich, we conducted a comparative cohort study including pediatric Long COVID patients. Microcirculation was assessed using sublingual sidestream dark field (SDF) imaging, analyzing the microvascular flow index (MFI), the total vessel density (TVD), and the proportion of perfused vessels (PPV). Endothelial function and arterial stiffness were evaluated using peripheral arterial tonometry (EndoPAT), measuring reactive hyperemia index (RHI) and augmentation index (AIx@75).

**Results:**

We analyzed 37 pediatric Long COVID patients (13.5 ± 2.6 years; 22 females) with persisting symptoms (> 4 weeks) and 46 healthy controls (12.4 ± 4.8 years; 21 females). Patients exhibited significant microcirculatory alterations, with reduced MFI (2.59 [IQR, 2.38–2.75] vs. 2.83 [IQR, 2.69–2.96]; p = .003), TVD (16.12 [IQR, 15.24–17.86] mm/mm2 vs. 19.38 [IQR, 17.58–20.57] mm/mm2; p < .001), and PPV (13.58 [IQR, 12.72–14.89]% vs. 17.67 [IQR, 16.60–19.32]%; p < .001). Microcirculatory changes varied with clinical phenotype and were most pronounced in patients presenting with dyspnea.We analyzed 37 pediatric Long COVID patients (13.5 ± 2.6 years; 22 females) with persisting symptoms (> 4 weeks) and 46 healthy controls (12.4 ± 4.8 years; 21 females). Patients exhibited significant microcirculatory alterations, with reduced MFI (2.59 [IQR, 2.38–2.75] vs. 2.83 [IQR, 2.69–2.96]; p = .003), TVD (16.12 [IQR, 15.24–17.86] mm/mm2 vs. 19.38 [IQR, 17.58–20.57] mm/mm2; p < .001), and PPV (13.58 [IQR, 12.72–14.89]% vs. 17.67 [IQR, 16.60–19.32]%; p < .001). Microcirculatory changes varied with clinical phenotype and were most pronounced in patients presenting with dyspnea.

*Conclusion*: We demonstrate measurable vascular alterations in pediatric Long COVID, including microvessel reduction and increased arterial stiffness. Our findings support a role of vascular changes in Long COVID and highlight the importance of integrating cardiovascular monitoring and follow-up into the management of affected children.

**Supplementary Information:**

The online version contains supplementary material available at 10.1007/s00431-026-06825-6.

**Table:** 

**What is Known:** • *Microvascular and endothelial dysfunction appear to play a role in SARS-CoV-2-related diseases.* • *Adults with Long COVID show persistent capillary rarefaction and endothelial impairment supporting a vascular mechanism underlying ongoing symptoms.*		
**What is New:** • *Pediatric Long COVID is likewise associated with significant microvascular damage and furthermore with increased arterial stiffness.* • *Children with dyspnea exhibit a distinct vascular phenotype characterized by marked capillary loss, indicating a potential microvascular origin of persistent respiratory symptoms.*		

## Introduction

While most patients recover rapidly from acute COVID-19, a significant proportion of 6–13% report persisting symptoms beyond the initial acute phase, including children [1–3]. These symptoms occur regardless of the severity of the initial disease and encompass a broad spectrum, including respiratory dysfunction, cardiovascular symptoms, persistent fatigue, and neurocognitive impairments [4]. Although some symptoms may appear mild in terms of clinical importance, they result in a substantial impairment of quality of life [1–3].

The terminology “Long COVID” and “post-COVID syndrome” rapidly emerged, later defined by the National Institute for Health and Care Excellence (NICE) [5]. According to this, Long COVID is defined by signs and symptoms that persist or develop after acute COVID-19, including both ongoing symptomatic COVID-19 (from 4 to 12 weeks) and post-COVID-19 syndrome (12 weeks or more) [5].

Although SARS-CoV-2 infection primarily manifests as a respiratory disease, many pathophysiological mechanisms occur at the vascular level [6]. Vascular effects, particularly microcirculatory impairment and endothelial dysfunction, have been observed in various SARS-CoV-2-related diseases including multisystem inflammatory syndrome in children (MIS-C) [7–9]. Potential pathophysiological mechanisms include direct viral toxicity, endotheliitis, microthrombosis, and immune system dysregulation with cytokine storms [10–13]. Such mechanisms may persist and contribute to pulmonary, cardiovascular, and neurological symptoms observed in Long COVID. Prolonged inflammation, either due to persistent SARS-CoV-2 infection or immune dysregulation, could further drive these effects [14–17]. Alterations in the microcirculation with persistent capillary rarefication and endothelial dysfunction have already been identified in adult patients with Long COVID [18–20].

In this study, we investigate (i) the general impact of Long COVID on microcirculation and endothelial function in a pediatric patient cohort and (ii) whether disease-specific clinical symptoms are associated with these microvascular and endothelial alterations.

## Methods

### Study design

This comparative cohort study included children with Long COVID, treated at the Children’s University Hospital of the LMU Munich, Germany, between December 2021 and February 2023. According to the study protocol, Long COVID patients were examined on the day of their initial presentation at our specialized multidisciplinary outpatient clinic for pediatric Long COVID patients. Both Long COVID patients and healthy controls were examined using the same SDF imaging system and the same EndoPAT device under identical conditions. Patients were examined in a single room without the presence of other patients or external distractions. The ambient temperature was maintained between 20 and 24 °C. Prior to the examination, patients were instructed to rest quietly in a supine position for 10 min. This ensures internal consistency and allows for valid comparative analysis of relative differences between groups.

This study was approved by the ethics committee of the medical faculty of Ludwig-Maximilian-University (LMU), Munich, Germany (Approval No. 22–0057) and carried out in accordance with the Declaration of Helsinki. We followed the STROBE guideline. The study is registered at the German Clinical Trials Register with the study protocol available online: https://drks.de/search/de/trial/DRKS00029642

### Participants

Children with serological evidence of a SARS-CoV-2 infection and a diagnosis of Long COVID according to NICE criteria, presenting with persisting symptoms for more than 4 weeks after confirmed infection, were eligible [5]. To exclude the potential influence of any undetected prior SARS-CoV-2 infection, we used a control group that had been evaluated prior to the pandemic between February 2018 and April 2019. The control group consisted of healthy children with a standardized clinical workup including uneventful clinical examination and no history of infectious diseases within the past 3 weeks. Exclusion criteria for both patients and controls included a history of hemato-oncologic, rheumatologic, or cardiac conditions as well as diabetes mellitus, hypercholesterolemia, and chronic inflammatory diseases (e.g., Crohn’s disease and ulcerative colitis). Controls showing any sign of inflammation, acute illness at the investigation time-point, medical conditions or requiring continuous medication were also excluded.

Written informed consent was obtained from all participants and from the parents or legal guardians prior to study participation.

### Procedures

#### Assessment of patients’ characteristics

Clinical patient characteristics were collected using a standard questionnaire addressing specific symptoms, pre-existing illnesses, routine clinical examinations (imaging and laboratory), and treatments. Weight, height, blood pressure, heart rate, and oxygen saturation were measured within 10 min before study examinations.

#### Assessment of the microcirculation

The sublingual microcirculation was visualized using a handheld video microscope (MicroScan, MicroVision Medical, Holland) with sidestream dark field (SDF) imaging technology. This microscope camera has an outer ring of greenlight-emitting diodes in the absorption maximum of hemoglobin (wavelength 530 nm) resulting in dark appearing vessels. The light penetrates the tissue and is reflected from deeper layers, enabling the transillumination of superficial layers. A central optical unit with a fivefold magnification lens detects the reflected light and generates a real-time video sequence of the microcirculation which is projected on a computer screen for later off-line analysis [21]. Recordings lasted 10 seconds each, with 3–10 attempts per patient depending on cooperation. To facilitate engagement, children could watch a cartoon if needed, and parents were allowed to sit beside them and provide support. Videos affected by movement artifacts (e.g., tongue or respiratory motion) were excluded, the three highest-quality recordings per patient were analyzed, and special care was taken to guarantee sufficient image recording quality [22]. All measurements were performed using the same camera and protocol as for the control cohort and processed with the automated AVA software without manual correction. The inter- and intrarater reliability of the measurement of the microcirculatory measurements using this system has previously been demonstrated [23].

Using the Automated Vascular Analysis program (AVA 3.2, MicroVision Medical Amsterdam, The Netherlands), we assessed total vessel density (TVD; mm/mm^2^), microvascular flow index (MFI; 0 = no flow, 1 = intermittent, 2 = sluggish, 3 = continuous flow), proportion of perfused vessels (PPV; %), and vessel diameter distribution (%). For statistical analyses, the mean of these recordings was used. Small vessels were defined as vessels with a diameter < 10 μm, medium vessels from ≥ 10 to < 25 μm, and large vessels ≥ 25 μm.

#### Assessment of endothelial function and arterial stiffness

The plethysmography EndoPAT device (EndoPAT, Itamar Medical Ltd., Israel) was used to evaluate endothelial function and arterial stiffness by measurement of the reactive hyperemia index (RHI) and the augmentation index normalized to a heart rate of 75 beats per minute (AIx@75, %), respectively [24]. The feasibility and reproducibility of this method in adolescents have been demonstrated previously [25].

These peripheral arterial tonometry (PAT) measurements were performed under a constant ambient temperature of 20–24 °C [26]. The examination consisted of 5 min baseline measurement pre-occlusion, 5 min arterial occlusion, and 5 min post-occlusion. Arterial occlusion was induced by inflating a blood pressure cuff placed around the upper arm to 60 mmHg above systolic pressure (max. 200 mmHg). Deflating the cuff leads to reactive hyperemia by arterial vasodilatation, which reflects local bioavailability of endothelial NO and represents a surrogate for endothelial function. The contralateral arm served as control to account for non-specific changes in vascular tone. An automated software program calculated the RHI and AI. Due to a temporary technical malfunction of the EndoPAT device, valid EndoPAT measurements could not be performed for all participants, particularly in the control group.

### Statistical analyses

Statistical analyses were performed with SPSS (IBM Corp. Released 2021. IBM SPSS Statistics for Windows, Version 29.0. Armonk, NY: IBM Corp), GraphPad Prism (Version 8.4.3.; GraphPad Software, LLC, San Diego, CA), and R (R Core Team. Released 2023. R for Windows, Version 4.3.3. Vienna, Austria: R Foundation for Statistical Computing).

As suitable microcirculatory data from children with COVID-19 were lacking, we based sample size calculation on preliminary control data from a previous study [26]. To detect a difference of 20% in means between patients and controls with a power of 0.8 at a significance level of 0.05 (dropout rate 0.1), we calculated required sample sizes ranging between 12 and 17 per group (see Suppl. Table 1).

Sex distribution of the groups was analyzed with the chi^2^-test. Normal distribution was tested using the Shapiro–Wilk test. For pairwise comparison, a T*-*Test or a Mann–Whitney *U* test was used as appropriate. When comparing three groups, a one-way ANOVA was employed with Tukey’s post hoc test for multiple comparisons. To confirm the results, we additionally conducted multivariate linear regression analysis adjusting for potential confounders, including age, BMI, blood pressure, and sex. A *p*-value < 0.05 was considered statistically significant.

Missing data in regression models were handled using complete case analysis (listwise deletion), as implemented by default in SPSS. Cases with missing values in any of the variables included in a given model were excluded from that specific analysis, and no imputation was applied. Assumptions of linear regression were assessed using standard diagnostic procedures in SPSS. Linearity and homoscedasticity were evaluated by visual inspection of residual plots, and normality of residuals was assessed using histograms and normal Q–Q plots, supplemented by the Shapiro–Wilk test. To assess the stability and robustness of the results, we performed bootstrap resampling of the control group with 5000 iterations, keeping the Long COVID group fixed. For each bootstrap sample, the same adjusted regression model was fitted, and the distribution of effect estimates was used to calculate mean beta estimates and 95% confidence intervals.

PCA was performed in R with the FactoMineR package. All data were standardized using the scale function to account for differing variable scales, and missing values were imputed using the imputePCA function from the missMDA package. The complete imputed dataset was used for subsequent analysis. Key outputs included eigenvalues, variance explained by each component, and variable contributions to the first two principal components (PC1 and PC2). Results were visualized using the factoextra package. Pairwise Spearman correlations were computed using the R cor function. The resulting correlation matrix was visualized with the corrplot package.

## Results

### Baseline clinical characteristics

We recruited 37 (22 female) patients with Long COVID at a mean of 206 ± 167 days following a positive SARS-CoV-2 test result. For the control group, we utilized data from 46 healthy children (21 female), who were part of a cohort examined in 2018. Demographic characteristics of the study cohort are displayed in Table 1. In the Long COVID cohort, reported pre-existing conditions included atopic dermatitis, asthma, anxiety disorder, diabetes insipidus, attention deficit hyperactivity disorder (ADHD), and persistent foramen ovale (PFO).

**Table 1:** Clinical characteristics of Long COVID patients and controls

Long COVID (*N* = 37)		Controls (*N* = 46)	*p*-value (*t*-test, *chi-square test)
Age, years	13.5 ± 2.6	12.4 ± 4.8	.190
Female sex, *N*	22	21	*.214
BMI, kg/m^2^	20.0 ± 4.7	18.6 ± 3.2	.127
SBP, mmHg	112 ± 11	114 ± 14	.438
DBP, mmHg	69 ± 6	68 ± 8	.428

Data are presented as mean ± SD. *T*-test was used for age, BMI, SBP, and DBP; chi^2^-test was used for sex, *BMI* body mass index, *DBP* diastolic blood pressure, *SBP* systolic blood pressure

At the time of assessment, anthropometric measurements and blood pressure were recorded for all participants.

**Fig. 1:**
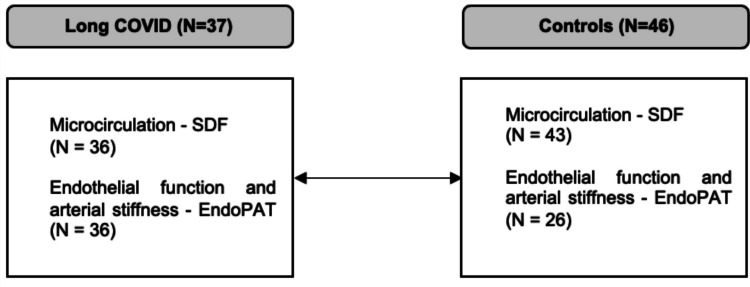
Number of patients and controls for each study examination

Due to technical reasons, it was not possible to obtain SDF or EndoPAT measurements in all children at that time. Figure 1 provides a detailed overview of the study groups.

All children with Long COVID reported at least one symptom related to their condition, with 33 (89%) experiencing multiple symptoms. The most frequently reported symptoms included headache (25 patients, 68%), reduced endurance (20 patients, 54%), fatigue (16 patients, 43%), and dyspnea (14 patients, 38%). Laboratory findings of the Long COVID cohort at the time of investigation are summarized in Suppl. Table 2. Overall, no significant abnormalities were detected, and in particular, inflammatory markers were not elevated.

### Microcirculation in Long COVID patients vs. controls

Long COVID patients differed significantly in microcirculatory parameters compared to healthy children, including significantly reduced MFI and TVD throughout the examined vessels, which in combination resulted in a generally lower PPV. The effect of Long COVID on microcirculatory parameters remained highly significant when adjusting for known confounders such as age, BMI, blood pressure, and sex (Table 2). Furthermore, our microcirculatory measurements revealed redistributed vessels in Long COVID patients with small-vessel rarefication, i.e., capillaries. Consecutively, medium-sized vessels and large vessels displayed a relative increase (Table 2).

**Table 2:** Microcirculation, endothelial function, and arterial stiffness in Long COVID patients and controls

Measure	Long COVID	Controls	Mann–Whitney *U* test	Multivariate linear regression		
			*p*-value^a^	*ß*^b^	95% CI	*p*-value^b^
SDF, *N*	36	43	NA	NA	NA	NA
MFI^c^ small vessels	2.66 (2.40 to 2.87)	2.92 (2.75 to 3.00)	<.001	−0.395	−0.122 to – 0.031	.001
MFI^c^ all vessels	2.59 (2.38 to 2.75)	2.83 (2.69 to 2.96)	<.001	−0.374	−0.121 to −0.027	.003
TVD small vessels, mm/mm^2^	4.61 (3.64 to 6.41)	9.53 (7.53 to 11.02)	<.001	− 0.683	− 1.761 to − 1.079	<.001
TVD all vessels, mm/mm^2^	16.12 (15.24 to 17.86)	19.38 (17.58 to 20.57)	<.001	− 0.630	− 1.285 to –0.697	<.001
PPV small vessels, %	4.00 (3.19 to 5.07)	9.21 (7.01 to 10.74)	<.001	− 0.735	− 1.791 to − 1.185	<.001
PPV all vessels, %	13.58 (12.72 to 14.89)	17.67 (16.60 to 19.32)	<.001	− 0.699	− 1.656 to –0.993	<.001
Small vessels^d^, %	29.97 (24.35 to 37.93)	49.08 (42.93 to 55.72)	<.001	− 0.675	− 7.217 to –4.390	<.001
Medium vessels^d^, %	56.59 (50.99 to 60.80)	45.81 (40.61 to 48.50)	<.001	0.455	1.617 to 4.090	<.001
Large vessels^d^, %	12.10 (9.74 to 17.99)	5.07 (3.50 to 7.68)	<.001	0.583	1.630 to 3.137	<.001
EndoPAT, *N*	36	26	NA	NA	NA	NA
RHI	1.43 (1.07 to 1.84)	1.20 (1.13 to 1.53)	.180	0.089	− 0.044 to 0.100	.439
AIx75	− 11.01 (− 18.15 to − 5.24)	− 21.29 (− 31.81 to − 11.91)	.001	0.329	0.747 to 4.709	.008

Data presented as median (IQR), *Aix75* Augmentation Index normalized for a heart rate of 75 bpm, *MFI* microvascular flow index, *NA* not applicable, *PPV* proportion of perfused vessels, *RHI* reactive hyperaemia index, *SDF* sidestream dark field, *TVD* total vessel density, *CI* confidence interval

^a^Mann-Whitney *U* test

^b^Multivariate linear regression adjusted for age, BMI, blood pressure, and sex

^c^MFI of 0 indicated no flow; 1, intermittent; 2, sluggish; and 3, continuous flow

^d^Vessel diameter distribution: Small vessels were defined as vessels with a diameter less than 10 μm; medium from 10 μm to less than 25 μm; and large 25 μm or larger

Regarding the EndoPAT measurements, we observed a significantly increased augmentation index (AIx@75) as a measure of arterial stiffness in Long COVID patients (Table 2).

To ensure that differences between the treatment group and the historic controls were not driven by time-related effects, we compared the historic control values with contemporary controls measured using the same protocol. The comparable results argue against temporal bias and support measurement reliability (Suppl. Table 3).

The pairwise comparisons of Long COVID patients versus controls gave nearly identical results between the original analysis and the bootstrap resampling. Likewise, bootstrap resampling yielded similar results to the multivariate regression (Suppl. Tables 4, and 5).

### Association of microcirculatory and clinical parameters

**Fig. 2:**
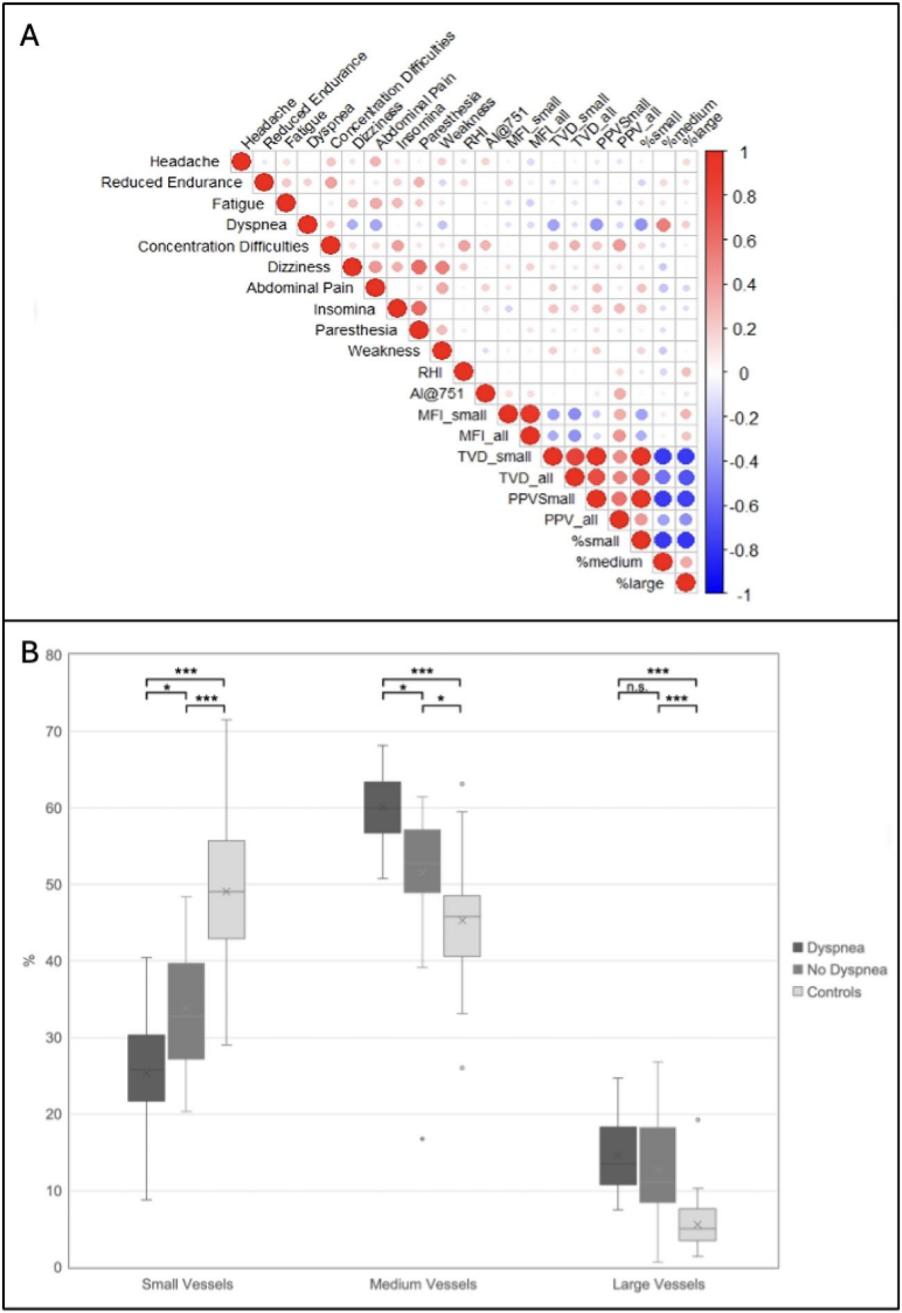
**A** Spearman correlation matrix of clinical and microcirculatory parameters from Long COVID patients. The Spearman correlation coefficient is color-coded, with values ranging from − 1 (indicated by blue) to + 1 (indicated by red). **B** Vessel distribution across Long COVID patients with and without dyspnea and controls. Small vessels were defined as vessels with a diameter less than 10 μm; medium, from 10 μm to less than 25 μm; and large, 25 μm or larger. *p*-values are derived from linear regression adjusted for age, BMI, blood pressure, and sex. One-way ANOVA with Tukey’s multiple comparison test: * =  < 0.05; *** < 0.001; n.s. = non significant

To explore whether specific Long COVID symptoms are associated with microvascular alterations or increased arterial stiffness, we visualized clinical and experimental data together in a correlation matrix (Fig. 2A). Overall, stronger associations were observed between the individual microcirculatory parameters than between the various Long COVID symptoms. There appears to be a positive association between dizziness, paresthesia, and a feeling of weakness. Notably, the symptom of dyspnea exhibited a divergent pattern compared to the other symptoms, showing an inverse correlation with small-vessel density and PPV small vessels.

Accordingly, when we stratified data from the Long COVID group by the presence of major symptom, patients with dyspnea demonstrated a further reduction in density of small vessels and an additional reduction in capillary density (Fig. 2B, Table 3).

**Table 3:** Vessel diameter distribution in Long COVID patients stratified by presence of dyspnea and controls and multivariate analysis of vessel diameter distribution

Measure	Dyspnea	No dyspnea	Controls	One-way ANOVA	Multivariate linear regression		
				*p*-value^a^	*ß*	95% CI	*p*-value^b^
Small vessels, %	25.82 (21.07 to 30.34)	32.79 (27.14 to 39.68)	49.08 (42.93 to 55.72)	<.001	− 0.638	− 7.72 to − 4.35	<.001
Medium vessels, %	59.97 (56.70 to 63.35)	52.79 (48.92 to 57.18)	45.81 (40.61 to 48.50)	<.001	0.398	1.29 to 4.19	<.001
Large vessels, %	13.52 (10.79 to 18.36)	11.24 (8.50 to 18.29)	5.07 (3.50 to 7.68)	<.001	0.607	1.86 to 3.55	<.001

Data presented as median (IQR). Small vessels were defined as vessels with a diameter less than 10 μm; medium from 10 μm to less than 25 μm; and large 25 μm or larger

*CI* confidence interval

^a^One way ANOVA

^b^Multivariate linear regression adjusted for age, BMI, blood pressure, and sex

Microvascular flow patterns and arterial stiffness, however, showed no significant differences related to the presence of dyspnea. Similarly, other symptoms, including headache, reduced endurance, fatigue, and dizziness, did not exhibit a significant impact on these vascular parameters.

We performed PCA on the full dataset to investigate the extent to which microcirculatory data could differentiate between Long COVID patients and controls. The first two principal components (PC1 and PC2) accounted for 52.8% and 19.2% of the total variance, respectively, cumulatively explaining 72.0% of the variability.

**Fig. 3:**
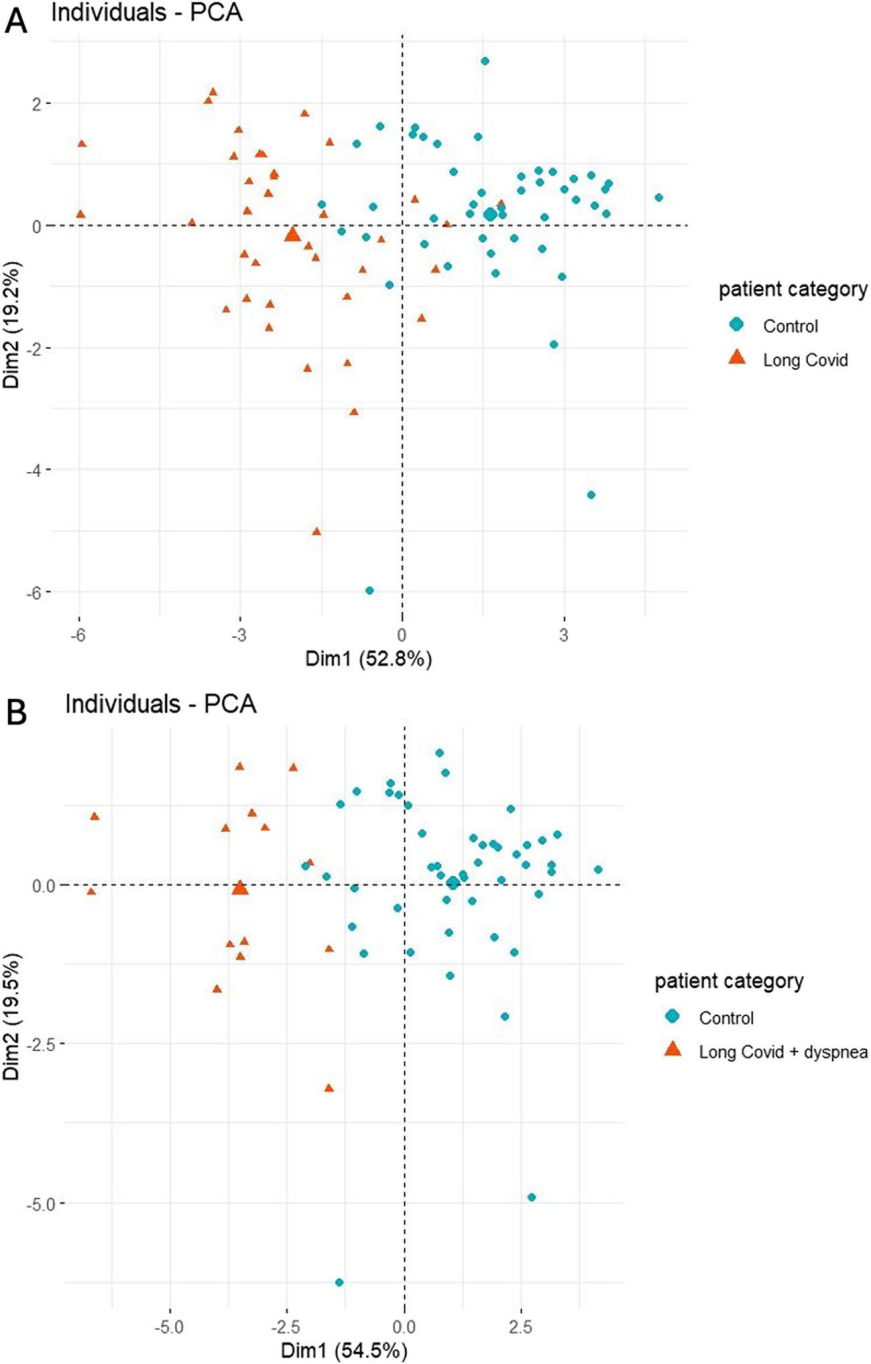
**A** Principal component analysis of microcirculatory data from Long COVID patients and controls. **B** Principal component analysis of microcirculatory data from Long COVID patients presenting with dyspnea and controls

The PCA scatterplot of individual scores revealed segregation of the two groups, with controls clustering predominantly on the right (higher PC1 values) and Long COVID patients distributed more broadly toward the left (Fig. 3A), indicating that Long COVID patients exhibit distinct microcirculatory phenotypes. PC1 was primarily driven by the PPV small (16.7%) and the TVD small (15.5%), while PC2 was mostly influenced by the MFI (38%).

Finally, when including only patients with dyspnea as a symptom in the PCA alongside controls, the Long COVID group now clustered more tightly along PC1 with only minimal overlap with the controls. This finding suggests that dyspnea is associated with a more distinct microcirculatory phenotype compared to other Long COVID manifestations, potentially reflecting consistent and specific pathophysiological changes within this subgroup (Fig. 3B).

## Discussion

In this study, we observed significant microvascular damage, including perfusion deficits, capillary loss, and increased arterial stiffness in pediatric Long COVID patients compared to healthy controls. Multivariate linear regression analyses, adjusted for age, BMI, blood pressure, and sex, confirmed Long COVID as an independent predictor of microcirculatory variables and arterial stiffness. Within the Long COVID group, patients with dyspnea exhibited additional impairment with capillary rarefication.

The pathophysiology of Long COVID remains incompletely understood and is the subject of ongoing research. Several mechanisms implicated in acute SARS-CoV-2 infection, such as endothelial inflammation and microcirculatory damage, may explain microvascular alterations observed in our cohort [10, 27–31]. Key mechanisms of Long COVID include viral persistence, post-acute inflammation, autoimmunity, thrombotic events, endothelial dysfunction, and mitochondrial dysfunction [32, 33]. These factors contribute to systemic immune dysregulation, tissue inflammation, metabolic disruption, and end-organ damage [32]. While most evidence to date originates from adult populations, recent pediatric studies also point to subtle but relevant endothelial changes [34].

Microvascular perturbations during the acute phase of COVID-19 have been reported in adults and children, including reduced capillary density, enlarged capillaries, and impaired perfusion observed by nailfold and conjunctival capillaroscopy [8, 35–38]. Some impairments may persist beyond the acute phase, consistent with endothelial dysfunction. In line with adult Long COVID studies demonstrating persistent capillary rarefication, our pediatric data suggest that similar microvascular alterations may already occur early in life [18, 19]. In a multivariate analysis, Charfeddine et al. identified endothelial dysfunction - assessed by finger thermal monitoring - along with female sex and severe clinical status during acute COVID-19 infection requiring oxygen supplementation as independent risk factors for Long COVID [20].

Similar long-lasting microvascular alterations have been described in MIS-C, supporting the concept that SARS-CoV-2-related vascular injury may persist beyond the acute phase [7].

Our findings underscore the involvement of the microvascular system and provide evidence for variability in vascular changes based on clinical symptoms. Specifically, patients with dyspnea exhibit more pronounced microvascular impairment compared to those with other symptoms. This suggests that the mechanisms underlying different phenotypes may vary significantly. Implementing microvascular monitoring in this heterogeneous patient population could offer valuable insights into the pathophysiology, aiding in the identification of Long COVID patients who may require intensified medical support and helping to mitigate the risk of severe complications in high-risk individuals.

The observed microcirculation damage and increased arterial stiffness in our pediatric Long COVID population may predispose affected children to a higher risk of cardiovascular disease such as hypertension and atherosclerosis in the long term. Whether these impairments persist over time and might contribute to sequelae - such as increased afterload leading to ventricular hypertrophy and heart failure, or insufficient tissue perfusion resulting in ischemia and organ dysfunction - remains speculative and requires more research. Nevertheless, our findings underscore the importance of understanding the cardiovascular implications of Long COVID.

This study has several limitations. First, the major limitation is the small study population caused by the rarity of diagnosed Long COVID cases in pediatric patients.

Second, the control group consists of historical controls recruited prior to the COVID-19 pandemic. This choice was a deliberate methodological decision to ensure that control participants had not been exposed to SARS-CoV-2. However, the use of historical controls may introduce temporal confounding and secular trends related to changes in environmental factors, health behaviors, or examination conditions over time. Sensitivity analyses including bootstrap resampling, as well as comparison with a small subgroup of contemporaneously examined controls, yielded consistent results. Nonetheless, residual temporal bias cannot be entirely excluded.

Third, the study may be subject to survivor bias and referral bias, as participants were recruited from our specialized Long COVID clinic. Individuals presenting to such clinics may differ systematically from the broader population of children and adolescents with post-acute sequelae of SARS-CoV-2 infection, and patients with milder or fully resolved symptoms may be underrepresented. In addition, the study population consisted predominantly of adolescents, reflecting the patient spectrum of our specialized pediatric Long COVID clinic and limiting the generalizability of the findings to younger children.

Furthermore, we assessed endothelial function and arterial stiffness using the EndoPAT device, which is designed for measurements in adults. Existing pediatric EndoPAT studies have generally included children > 10 years and adolescents, while the youngest child in our study was 6 years old [39]. This may potentially cause problems by a lack of cooperation during measurements as arterial occlusion for 5 min may be uncomfortable, and the fingertip probes may not fit properly. As raw post-occlusive data were not available, peak-based analyses of maximal dilation could not be performed, representing a further methodological limitation. Together with the small number of control patients with valid EndoPAT data, this may have reduced the validity and statistical power of our analyses.

Physical activity levels and cardiorespiratory fitness were not quantitatively assessed and therefore could not be included in baseline characteristics or statistical analyses. Reduced physical activity and exercise intolerance are common in pediatric Long COVID and may independently impair endothelial function and increase arterial stiffness, which should be considered when interpreting the findings. Prolonged reductions in physical activity can independently affect endothelial function and vascular stiffness [40].

Follow-up measurements were not feasible due to considerable travel distances and symptom resolution in a relevant portion of patients.

Lastly, it would be of great interest to compare microvascular function and arterial stiffness in children with Long COVID to those who were infected with SARS-CoV-2 but did not develop persistent symptoms to isolate the specific effects of Long COVID more precisely. However, recruiting such asymptomatic post-COVID patients is challenging, as they typically do not present to the hospital. Therefore, it remains unclear whether the observed microvascular alterations are specifically associated with Long COVID or represent a general consequence of SARS-CoV-2 infection.

## Conclusion

Our findings collectively reveal substantial microvascular impairments and increased arterial stiffness in pediatric Long COVID patients, particularly among those presenting with dyspnea. These results are concerning and point to the potential involvement of disease-specific pathophysiological mechanisms. However, further longitudinal studies involving larger patient cohorts and extended follow-up periods are essential to comprehensively characterize the trajectory of these abnormalities and to uncover the underlying mechanisms. Monitoring microcirculatory function and endothelial health in these patients could provide valuable biomarkers, critical not only for optimizing the clinical management but also for informing the development of targeted therapeutic strategies and novel interventions for Long COVID.

## Supplementary Information

Below is the link to the electronic supplementary material.

ESM 1 (DOCX 29.9 KB)

ESM 2 (DOCX 17.6 KB)

ESM 3 (DOCX 17.8 KB)

ESM 4 (DOCX 16.7 KB)

ESM 5 (DOCX 15.4 KB)

## Data Availability

Individual participant data that underlies the results reported in this article, after de-identification are available upon request.

## Abbreviations

ADHD: Attention deficit hyperactivity disorder
AIx@75: Augmentation index normalized to a heart rate of 75 bpm
AVA: Automated Vascular Analysis
BMI: Body mass index
CRP: C-reactive protein
DBP: Diastolic blood pressure
ESR: Erythrocyte sedimentation rate
IgG: Immunoglobulin G
IQR: Interquartile range
LDH: Lactate dehydrogenase
MFI: Microvascular flow index
MIS-C: Multisystem inflammatory syndrome in children
NO: Nitric oxide
NICE: National Institute for Health and Care Excellence
OCT: Optical coherence tomography
PAT: Peripheral arterial tonometry
PC: Principal component
PCA: Principal component analysis
PFO: Persistent foramen ovale
PPV: Proportion of perfused vessels
RHI: Reactive hyperemia index
SARS-CoV-2: Severe acute respiratory syndrome coronavirus 2
SDF: Sidestream dark field
SD: Standard deviation
SBP: Systolic blood pressure
STROBE: Strengthening the Reporting of Observational Studies in Epidemiology
TVD: Total vessel density
