# Supplementary material for: Microcirculatory impairment and increased arterial stiffness in pediatric Long COVID patients
Source: Eur J Pediatr. 2026 Mar 16;185(4):186. doi: 10.1007/s00431-026-06825-6 (PMC12992436; doi:10.1007/s00431-026-06825-6)
Supplement: Supplementary file 1 — (DOCX 29.9 KB) [file 431_2026_6825_MOESM1_ESM.docx]

| Parameter | Median | SD | Δ (20%) | P(X>Y) (approx.) | Required sample size (per group) |
| --- | --- | --- | --- | --- | --- |
| RHI | 1.32 | 0.22 | 0.26 | 0.80 | 17 |
| TVD | 19.47 | 1.85 | 3.89 | 0.86 | 13 |
| MFI | 2.83 | 0.38 | 0.57 | 0.83 | 15 |

Suppl. Tab. 1: Sample size calculation using a non-parametric Wilcoxon test based on data from [1].

Δ = 20% difference between groups; power = 0.8; α = 0.05; dropout rate 0.1.

1. B. Pastor-Villaescusa, J. Meier, F. Ruske, C. Prell, J. Gruenzner, M. Koenig, A. Jakob, B. Koletzko and C. Nussbaum. 2024. Article Association between Inflammation, Glycocalyx Biomarkers, and Endothelial Function in Children with Hypercholesterolemia. Journal. 80(Issue): 260-267.
