# Supplementary material for: Microcirculatory impairment and increased arterial stiffness in pediatric Long COVID patients
Source: Eur J Pediatr. 2026 Mar 16;185(4):186. doi: 10.1007/s00431-026-06825-6 (PMC12992436; doi:10.1007/s00431-026-06825-6)
Supplement: Supplementary file 2 — (DOCX 17.6 KB) [file 431_2026_6825_MOESM2_ESM.docx]

| **Laboratory Parameter** | **Unit** | **N** | **Mean ± Standard Deviation** |
| --- | --- | --- | --- |
| Hemoglobin | g/dl | 31 | 14.0 ± 2.0 |
| Leukocytes | G/l | 31 | 6.1 ± 1.2 |
| Neutrophils | G/l | 31 | 3.2 ± 0.8 |
| Lymphocytes | G/l | 31 | 2.1 ± 0.5 |
| Platelets | G/l | 31 | 286.4 ± 76.1 |
| C-Reactive Protein, CRP | mg/dl | 26 | 0.1 ± 0.0 |
| Ferritin | ng/ml | 17 | 65.2 ± 33.1 |
| Erythrocate Sedimentation Rate, ESR | mm/h | 22 | 7.1 ± 5.2 |
| Immunoglobulin G, IgG | g/l | 18 | 10.9 ± 2.0 |
| Lactate dehydrogenase, LDH | U/l | 26 | 191.7 ± 47.7 |

Suppl. Tab. 2: Laboratory Parameters in Children with Long COVID.

Data are presented as mean ± SD.
