# Supplementary material for: Microcirculatory impairment and increased arterial stiffness in pediatric Long COVID patients
Source: Eur J Pediatr. 2026 Mar 16;185(4):186. doi: 10.1007/s00431-026-06825-6 (PMC12992436; doi:10.1007/s00431-026-06825-6)
Supplement: Supplementary file 3 — (DOCX 17.8 KB) [file 431_2026_6825_MOESM3_ESM.docx]

|  | **Controls (N=43)** | **Contemporary healthy controls (N=3)** | **Controls (N=43)** | **Contemporary healthy controls (N=3)** | **MWU** |
| --- | --- | --- | --- | --- | --- |
|  | Mean ± SD | Mean ± SD | Median (IQR) | Median (IQR) | p-value |
| TVD all vessels, mm/mm^2^ | 19.0 ± 2.0 | 19.2 ± 0.1 | 19.4 (11.9 – 22.9) | 19.2 (19.1 – 19.3) | .737 |
| MFI all vessels^a^ | 2.8 ± 0.3 | 2.8 ± 0.0 | 2.8 (1.5 – 3.0) | 2.8 (2.8 – 2.8) | .582 |
| PPV all vessels, % | 17.6 ± 2.4 | 17.8 ± 0.1 | 17.7 (10.4 – 21.2) | 17.8 (17.7 – 17.8) | .900 |

Suppl. Tab. 3: Comparison of Microvascular Parameters Between Study and Contemporary Healthy Controls.

Data presented as mean ± SD and median (IQR). Abbreviations: MFI, Microvascular Flow Index; PPV, Portion of Perfused Vessels;; TVD, Total Vessel Density. Statistical comparisons using the Mann–Whitney-U-test should be interpreted with caution due to the small sample size of the contemporary control group (N = 3).

^a^MFI of 0 indicated no flow; 1, intermittent; 2, sluggish; and 3, continuous.
