# Supplementary material for: Microcirculatory impairment and increased arterial stiffness in pediatric Long COVID patients
Source: Eur J Pediatr. 2026 Mar 16;185(4):186. doi: 10.1007/s00431-026-06825-6 (PMC12992436; doi:10.1007/s00431-026-06825-6)
Supplement: Supplementary file 4 — (DOCX 16.7 KB) [file 431_2026_6825_MOESM4_ESM.docx]

| **Measure** | **Long COVID** | **Controls** | **Mann-Whitney-U-Test p-value** | **ß** | **95% CI** | **p-value (multivariable)** |
| --- | --- | --- | --- | --- | --- | --- |
| MFI small vessels | 2.66 (2.40 to 2.85) | 2.92 (2.75 to 3.00) | <.001 | -0.229 | -0.366 to -0.092 | 0.001 |
| MFI all vessels | 2.59 (2.38 to 2.73) | 2.83 (2.69 to 2.96) | <.001 | -0.222 | -0.363 to -0.081 | 0.003 |
| TVD small vessels, mm/mm^2^ | 4.61 (3.68 to 6.32) | 9.53 (7.64 to 10.90) | <.001 | -4.26 | -5.283 to -3.238 | <.001 |
| TVD all vessels, mm/mm^2^ | 16.12 (15.35 to 17.82) | 19.38 (17.65 to 20.42) | <.001 | -2.974 | -3.855 to -2.092 | <.001 |
| PPV small vessels, % | 4.00 (3.25 to 5.06) | 9.21 (7.19 to 10.47) | <.001 | -4.455 | -5.365 to -3.544 | <.001 |
| PPV all vessels, % | 13.58 (12.75 to 14.89) | 17.67 (16.61 to 19.29) | <.001 | -3.973 | -4.967 to -2.978 | <.001 |
| Small vessels, % | 29.97 (24.97 to 37.48) | 49.08 (43.40 to 55.32) | <.001 | -17.4 | -21.636 to -13.164 | <.001 |
| Medium vessels, % | 56.59 (51.40 to 60.66) | 45.81 (40.64 to 48.45) | <.001 | 8.56 | 4.851 to 12.269 | <.001 |
| Large vessels, % | 12.10 (9.91 to 17.93) | 5.07 (3.52 to 7.41) | <.001 | 7.151 | 4.891 to 9.412 | <.001 |
| RHI | 1.42 (1.08 to 1.82) | 1.20 (1.10 to 1.59) | 0.233 | 0.08 | -0.146 to 0.307 | 0.481 |
| AIx75 | -10.95 (-18.04 to -5.32) | -23.59 (-31.81 to -17.28) | <.001 | 9.752 | 3.829 to 15.676 | 0.002 |

Suppl. Tab. 4: Bootstrap resampling of the control group with 5,000 iterations, keeping the Long COVID group fixed.

Data presented as median (IQR). Abbreviations: Aix75, Augmentation Index normalized for a heart rate of 75 beats/min; MFI, Microvascular Flow Index; PPV, Portion of Perfused Vessels; RHI, Reactive Hyperaemia Index; SDF, sidestream dark field; TVD, Total Vessel Density.

MFI of 0 indicated no flow; 1, intermittent; 2, sluggish; and 3, continuous; ^d^ Vessel diameter distribution: Small vessels were defined as vessels with a diameter less than 10 μm; medium, from 10 μm to less than 25 μm; and large, 25 μm or larger.

CI: Confidence Interval
