# Supplementary material for: Microcirculatory impairment and increased arterial stiffness in pediatric Long COVID patients
Source: Eur J Pediatr. 2026 Mar 16;185(4):186. doi: 10.1007/s00431-026-06825-6 (PMC12992436; doi:10.1007/s00431-026-06825-6)
Supplement: Supplementary file 5 — (DOCX 15.4 KB) [file 431_2026_6825_MOESM5_ESM.docx]

| **Measure** | **No Dyspnea** | **Dyspnea** | **Controls** | **One-way ANOVA p-value** | **ß (multivariate)** | **95% CI (multivariate)** | **ß (bootstrap)** | **95% CI (bootstrap)** | **p-value (multivariate)** |
| --- | --- | --- | --- | --- | --- | --- | --- | --- | --- |
| Small% | 25.82 (21.07 to 30.34) | 32.79 (27.14 to 39.68) | 49.08 (42.93 to 55.72) | <.001 | -0.638 | -7.72 to -4.35 | -0.6 | -7.52 to -3.98 | <.001 |
| Medium% | 59.97 (56.70 to 63.35) | 52.79 (48.92 to 57.18) | 45.81 (40.61 to 48.50) | <.001 | 0.398 | 1.29 to 4.19 | 0.41 | 1.15 to 4.60 | <.001 |
| Large% | 13.52 (10.79 to 18.36) | 11.24 (8.50 to 18.29) | 5.07 (3.50 to 7.68) | <.001 | 0.607 | 1.86 to 3.55 | 0.615 | 1.76 to 3.45 | <.001 |

Suppl. Tab. 5: Bootstrap resampling of the control group with 5,000 iterations.

Data presented as median (IQR).

Vessel diameter distribution: Small vessels were defined as vessels with a diameter less than 10 μm; medium, from 10 μm to less than 25 μm; and large, 25 μm or larger.

CI: Confidence Interval
